# The soluble transhydrogenase UdhA affecting the glutamate-dependent acid resistance system of *Escherichia coli* under acetate stress

**DOI:** 10.1242/bio.031856

**Published:** 2018-09-15

**Authors:** Hanjun Zhao, Feng Zhou, Quan Xing, Zhengyu Cao, Jie Liu, Guoping Zhu

**Affiliations:** The Research Center of Life Omics and Health, College of Life Sciences, Anhui Normal University, No.1 Beijing East Road, Wuhu 241000, Anhui, China

**Keywords:** Acid resistance, *Escherichia coli*, NADH, Transhydrogenase, UdhA

## Abstract

The soluble transhydrogenase (UdhA) is one of two transhydrogenases that play a role in maintaining the balance between NAD(H) pools and NADP(H) pools in *Escherichia coli*. Although UdhA has been extensively used in metabolic engineering and biocatalysis for cofactor regeneration, its role in acid resistance has not been reported. Here we used DNA microarray to explore the impact of UdhA on transcript levels. We demonstrated that during growth on acetate, the expression of genes involved in the respiratory chain and Gad acid resistance system was inhibited in the *udhA*-knockout strain. The deletion of *udhA* significantly repressed the expression of six genes (*gadA, gadB, gadC, gadE, hdeA* and *hdeB*) which are involved in Gad acid resistance and resulted in low survival of the bacterium at a low pH of 4.9. Moreover, UdhA was essential for NADH production which is important for the adaptive growth of *E. coli* on acetate, while NADH concentration in the *udhA*-knockout strain was quite low and supplemental NADH significantly increased the expression of acid resistance genes and survival of the *udhA*-knockout strain. These results demonstrated that UdhA is an important source of NADH of *E. coli* growth on acetate and affects Gad acid resistance system under acetate stress.

## Abstract (precis)

**Summary:** UdhA function stated in this study helps us to understand the physiological roles of UdhA affecting NADH production and Gad acid resistance system in *E.coli* in acetate environment.

## INTRODUCTION

To pass through the stomach and survive in the intestine, enteric pathogens have evolved a number of strategies for adaptation to extremely acidic environments. For instance, *Escherichia coli* O157:H7, a particularly virulent form of *E. coli,* can shift from the nurturing pH 7 environment of a hamburger to the harsh pH 2 milieu of the stomach within moments (Foster, 2004). This organism is a highly acid-resistant food-borne pathogen (Foster, 2004; Price et al., 2004). Comparison studies have revealed that enterohemorrhagic *E. coli* O157:H7 (pathogenic) and commensal *E. coli* (non-pathogenic) possess similar acid resistance (AR) systems, conferring upon them equally outstanding capabilities to overcome acidic barriers (Foster, 1991, 2004; Price et al., 2004).

Four acid resistance systems have been identified in *E. coli* (Foster, 2004; Stincone et al., 2011; Sun et al., 2011). The first system (AR1), which is poorly understood, is active in the absence of amino acids (Lin et al., 1996), requires the sigma factor RpoS (Castanie-Cornet et al., 1999; Price et al., 2000) and the catabolite repressor protein CRP (Castanie-Cornet and Foster, 2001), and consumes ATP (Sun et al., 2011). The other three systems are dependent on the external supply of amino acids and are composed of dedicated pairs of amino acid decarboxylases and antiporters (Foster, 2004). The second system (AR2), which is the most effective glutamate-dependent system, involves two glutamate decarboxylase isozymes (GadA and GadB) and a putative glutamate/γ-amino butyric acid antiporter (GadC) (Castanie-Cornet et al., 1999). The third system (AR3), which is an arginine-dependent system, requires arginine decarboxylase (AdiA) and an arginine/agmatine antiporter (AdiC) (Castanie-Cornet et al., 1999; Gong et al., 2003; Iyer et al., 2003). The fourth system (AR4), which is a lysine-dependent, but much less efficient system, relies on lysine decarboxylase (CadA) and a lysine/cadaverin antiporter (CadB) (Iyer et al., 2003; Meng and Bennett, 1992; Vazquez-Juarez et al., 2008). The ARs hold up an ‘umbrella’ that protects *E. coli* under a variety of different acid stress situations. Moreover, it has been reported that many regulator proteins are directly or indirectly involved in ARs, such as CysB, EvgA/EvgS, GadE, GadX, GadW, HU, SspA and YdeO (Bi et al., 2009; Foster, 2004; Hansen et al., 2005; Lochowska et al., 2004; Masuda and Church, 2002; Sayed et al., 2007; Tramonti et al., 2006, 2002), and that ATP is required for survival under extremely acidic conditions (Sun et al., 2011).

The model organism *E. coli*, a typical Gram-negative bacterium, has been used to represent the group of enteric pathogens (such as *Salmonella* and *Campylobacter*). Although the genomes of various *E. coli* strains have been sequenced since 1997 (Blattner et al., 1997; Latif et al., 2014; Monk et al., 2013; Yoon et al., 2012), the physiological roles of many genes are still unclear, such as a soluble pyridine nucleotide transhydrogenase (STH). STH is only found in Gram-negative enteric bacteria, Actinomycetes and some other bacteria (Boonstra et al., 2000a). The soluble transhydrogenase, encoded by *udhA* in *E. coli*, is an energy-independent flavoprotein and forms remarkably large polymers (Boonstra et al., 2000a). Several experiments suggest that UdhA plays a role in reoxidizing excess NADPH into NADP and transferring the hydrogen (H) electron to NADH (Boonstra et al., 2000b; Canonaco et al., 2001; Sauer et al., 2004; Voordouw et al., 1983). Also, STH has been extensively employed for applications in metabolic engineering and biocatalysis. For example, STH from *Pseudomonas fluorescens* has been used in a cell-free system for efficient coenzyme cycling, resulting in high yields of hydromorphone, a semisynthetic opiate (Boonstra et al., 2000b). However, despite these technological advances, the functions of UdhA *in vivo* remain obscure.

In this study, DNA microarrays and mutant strains were used to explore the physiological roles of UdhA in *E. coli*. Our developing model implies that UdhA is an important source of NADH for the adaptive growth of *E. coli* on acetate and affects Gad acid resistance system of *E. coli* under acetate stress.

## RESULTS

### Growth rates of wild-type and mutant *E. coli*

The growth rates of ZG2 (*icdA*^NAD^, *E. coli* containing an engineered NAD^+^-dependent isocitrate dehydrogenase), ZG3 (*icdA*^NADP^Δ*udhA*, *udhA*-knockout *E. coli* containing NADP^+^-dependent isocitrate dehydrogenase) and ZG4 (*icdA*^NAD^Δ*udhA*, *udhA*-knockout *E. coli* containing an engineered NAD^+^-dependent isocitrate dehydrogenase) were similar to that of the wild-type ZG1 (*icdA*^NADP^, wild-type *E. coli* containing NADP^+^-dependent isocitrate dehydrogenase) when glucose was the sole source of carbon and energy (Zhao et al., 2008). The growth rate of ZG3 on acetate was significantly lower than that of ZG1. The growth rate of ZG4 was recovered when the coenzyme specificity of isocitrate dehydrogenase (IDH) was changed from NADP-dependency to NAD-dependency (Table 1 and Table 2).

**Table 1.**
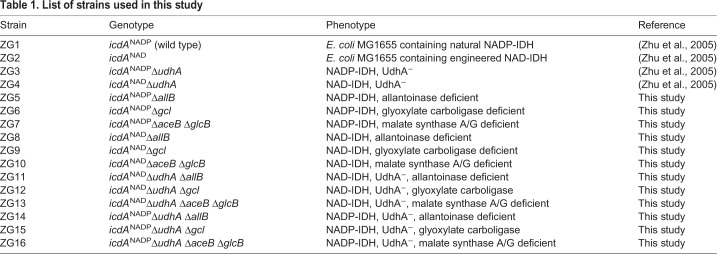
**List of strains used in this study**

**Table 2.**
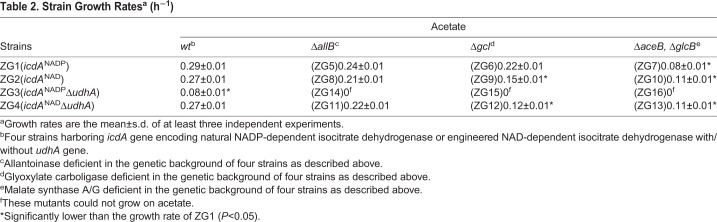
**Strain Growth Rates^a^ (h^−1^)**

### Transcriptome analysis

To investigate the poor growth rate of ZG3 on acetate, the transcript profiles of the wild-type strain ZG1 and the mutant strains ZG2, ZG3 and ZG4 were performed during growth on acetate. The total RNAs of all strains were collected at the time of OD_600_=0.6. Transcriptome analysis showed that the differences in the expression of most genes were small (<twofold) and were not statistically significant. The most significant differences were reductions in expression observed in the mutants (Table 3). Genes involved in the respiratory chain and acid resistance system were downregulated in ZG3. In addition, four genes involved in glyoxylate metabolism were upregulated in ZG2 and ZG4. To confirm the gene expression data from the DNA microarray, reverse transcription polymerase chain reaction (RT-PCR) confirmed that compared with the wild-type ZG1, the transcript levels of NADH dehydrogenase-2 (NDH-2, encoded by *ndh*) and isocitrate dehydrogenase (IDH, encoded by *icdA*) were similar to those obtained through microarray results (Fig. S1).

**Table 3.**
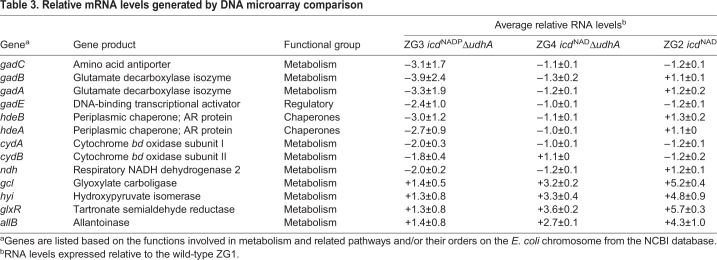
**Relative mRNA levels generated by DNA microarray comparison**

### Genes involved in acid resistance

**Fig. 1.**
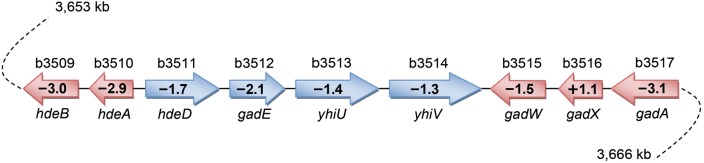
**The cluster of nine genes involved in the acid resistance system in the *E. coli* genome.** The arrows represent the genes and the direction of transcription. Gene names are shown below the gene, and their entry numbers are shown above the gene. The fold-change of each gene in ZG3 (*icdA*^NADP^Δ*udhA*) is provided inside the arrows.

During growth on acetate, the expression of six genes involved in AR2 (*gadA, gadB, gadC, gadE, hdeA* and *hdeB*) was evidently reduced in ZG3 compared with wild-type ZG1 (Table 3). The *gadA* and *gadB* genes encode isoforms of glutamate decarboxylase, and *gadC* encodes a γ-aminobutyrate (GABA) antiporter (Castanie-Cornet et al., 1999, 2007). The GadC antiporter together with the GadA and GadB decarboxylases maintain the intracellular pH in cells subjected to extreme acid stress (Arnold et al., 2001). The product of *gadE* regulates the acid-induced expression of *gadA, gadB* and *gadC* (Ma et al., 2003). In addition, *hdeA* and *hdeB* encode two acid stress chaperones that prevent periplasmic protein aggregation at low pH (Kern et al., 2007). Four genes, *gadA*, *gadE*, *hdeA* and *hdeB*, cluster with *gadW*, *gadX*, *hdeD*, *yhiU* and *yhiV* between 3650 and 3666 kb in the *E. coli* genome (Fig. 1). The expression of each gene except for *gadX* was reduced to some extent in ZG3 (Table 3 and Fig. 1). In all, eleven genes (including the *gadBC* operon) involved in bacterial acid resistance were repressed.

### Acid resistance assays

**Fig. 2.**
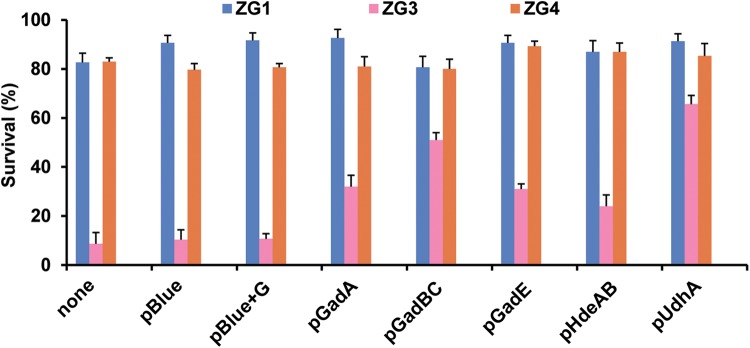
**Survival of acid-challenged strains.** The percentage survival of strains challenged at pH 4.9 for 1 h was calculated compared with the count at time zero*.* Plasmids are denoted below the x-axis: none, no plasmid; pBlue, empty vector; pBlue+G, empty vector with glutamate in the medium, pGadA, pGadBC, pGadE, pHdeAB and pUdhA. IPTG and glutamate were supplemented in the culture media. The survival of ZG3 (*icdA*^NADP^Δ*udhA*) was significantly increased (*P*<0.05) by the expression of pHdeAB, pGadA, pGadBC, pGadE or pUdhA compared with ZG3 with or without pBlue. The survival of ZG1 (*icdA*^NADP^, WT) and ZG4 (*icdA*^NAD^Δ*udhA*) was not significantly affected by the plasmids (*P*>0.05). Data are presented as the mean±s.d. of three independent experiments.

To verify whether ZG3 was sensitive to the acid stress which may be caused by reduced expression of acid resistance genes, the genes including *udhA*, *gadA*, the *gadBC* operon, *gadE* and the *hdeAB* operon were cloned and expressed in various genetic backgrounds. It was found that 80% of ZG1 and ZG4 cells, either with or without plasmids, survived for 1 h at pH 4.9 (Fig. 2). By contrast, only 9% of ZG3 cells survived. In the presence of pUdhA, ZG3 survival was increased to 66%. Expressing acid-resistance genes such as *gadA*, the *gadBC* operon, *gadE* or the *hdeAB* operon also improved ZG3 survival, although not as strongly as pUdhA (Fig. 2). However, the survival of ZG3 harboring pBlue was not increased, regardless of whether glutamate was present. These results indicated that UdhA plays an important role in affecting the Gad acid resistance system of *E. coli.*

### Respiratory chain genes

The aerobic respiratory chain of *E. coli* is composed of a number of dehydrogenases and two quinol oxidase complexes. The microarray data showed that during growth on acetate, no significant changes occurred in the expression of either the *sdhCDAB* operon, which encodes succinate dehydrogenase, or the *cyoABCDE* operon, which encodes the cytochrome *bo* quinol oxidase (data not shown). Only the expression of *ndh*, which encodes a NADH dehydrogenase (NDH-2), and the *cydAB* operon, which encodes the heterodimeric cytochrome *bd* quinol oxidase (Zhang et al., 2004), were reduced twofold in ZG3 (Table 3).

### Genes involved in glyoxylate metabolism

When acetate is the sole available source of carbon and energy for *E. coli*, it is first activated to acetyl-CoA and then metabolized through the tricarboxylic acid cycle and glyoxylate shunt (Oh et al., 2002). Genes (*allB*, *gcl* and *glxR*) involved in glyoxylate metabolism (Cusa et al., 1999) showed increased expression in ZG2 and ZG4 (Table 3). Gene *allB*, which encodes the allantoinase that converts allantoin to allantoate, was upregulated by 4.3-fold in ZG2 and 2.7-fold in ZG4. Gene *gcl*, which encodes glyoxylate carboligase, and *glxR*, which encodes tartronate semialdehyde reductase, were upregulated by 5.2-fold and 5.7-fold in ZG2 and by 3.2-fold and 3.6-fold in ZG4, respectively. In addition, *hyi*, located downstream of *glxR*, encodes hydroxypyruvate isomerase and was upregulated 4.8-fold in ZG2 and 3.3-fold in ZG4.

### Growth of deletion strains

**Fig. 3.**
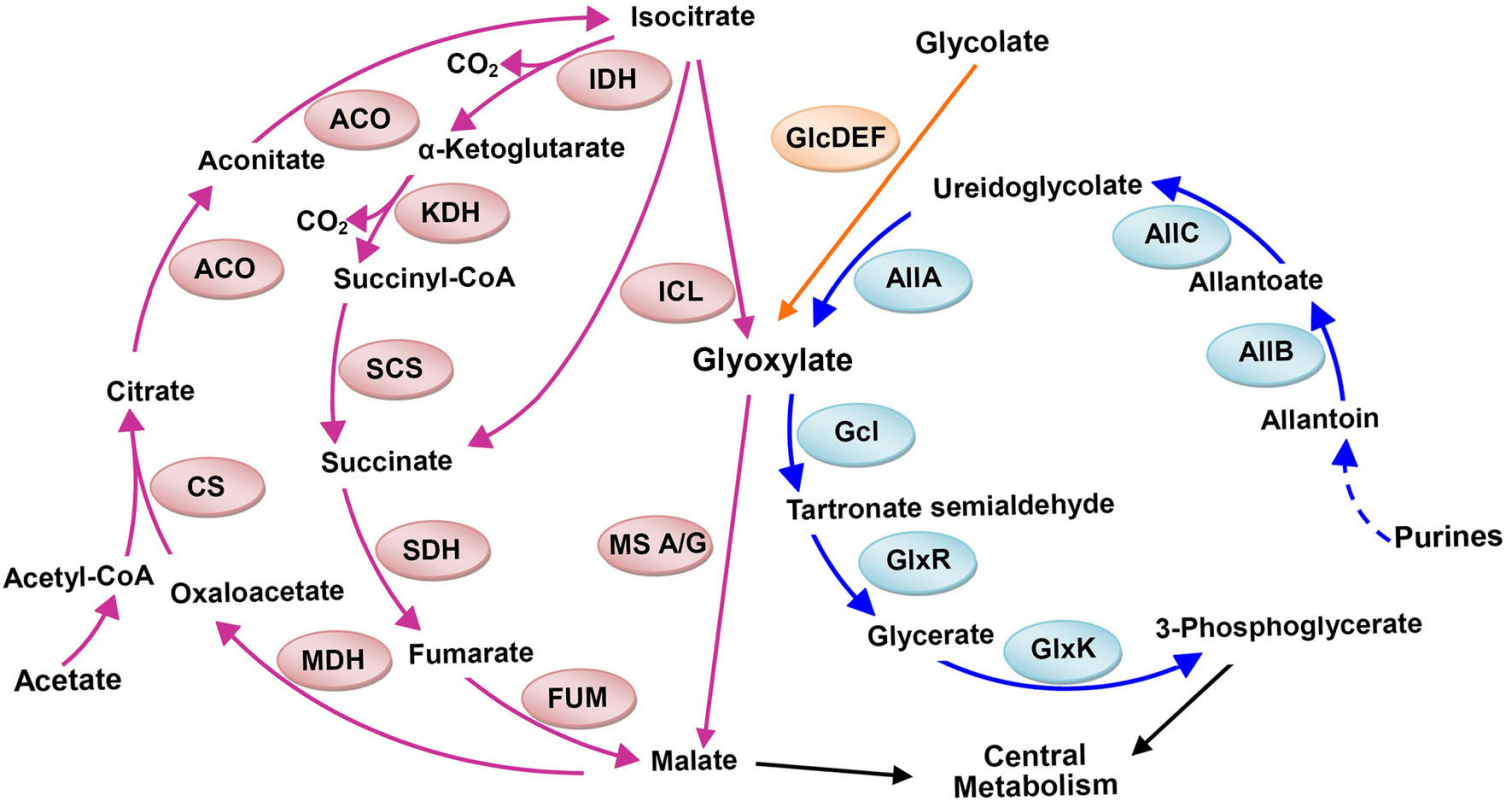
**Pathways involved in glyoxylate metabolism.** Three pathways generate glyoxylate: glycolate metabolism, acetate metabolism and purine degradation. Glyoxylate enters the central metabolism through two pathways: direct conversion to malate by malate synthase G (GlcB) and/or malate synthase A (AceB) or conversion to 3-phosphoglycerate by glyoxylate carboligase (Gcl), tartronate semialdehyde reductase (GlxR) and glycerate kinase (GlxK). Genes with blue backgrounds were upregulated in ZG2 (*icdA*^NAD^) and ZG3 (*icdA*^NADP^Δ*udhA*). ACO, aconitase; AllB, allantoinase; AllC, allantoate amidohydrolase; AllA, ureidoglycolate hydrolase; CS, citrate synthase; FUM, fumarase; Gcl, glyoxylate carboligase; GlxK, glycerate kinase; GlxR, tartronic semialdehyde reductase; ICL, Isocitrate lyase; KDH, α-Ketoglutarate dehydrogenase; MDH, malate dehydrogenase; SCS, Succinyl-CoA synthetase; SDH, succinate dehydrogenase.

Nine mutants (Table 1) were constructed to explore how deleting *udhA* and changing IDH coenzyme specificity influence the glyoxylate metabolism pathways (Fig. 3). Without allantoinase (encoded by *allB*), glyoxylate carboligase (encoded by *gcl*) or malate synthase A/G (encoded by *aceB* and *glcB*), the growth rates of all constructed strains (ZG5-ZG16) were lower than that of ZG1, ZG2, ZG3 and ZG4, correspondingly (Table 2). Deleting *allB* had the least effect on the growth rate. Deleting *gcl* further reduced the growth rates, particularly in ZG9 (*icdA*^NAD^Δ*gcl*) and ZG12 (*icdA*^NAD^/Δ*udhA* Δ*gcl*). The greatest reductions in the growth rate were observed when *aceB* and *glcB* in the glyoxylate bypass were deleted. Surprisingly, ZG7 (*icdA*^NADP^Δ*aceB* Δ*glcB*) grew more slowly on acetate than either ZG10 (*icdA*^NAD^Δ*aceB*Δ*glcB*) or ZG13 (*icdA*^NAD^Δ*udhA* Δ*aceB* Δ*glcB*). These results indicated that glyoxylate metabolism played an important role in *E. coli* during growth on acetate, which was probably regulated by the NADH pool as identified in followings.

### The effect of NADH on growth and acid resistance

**Fig. 4.**
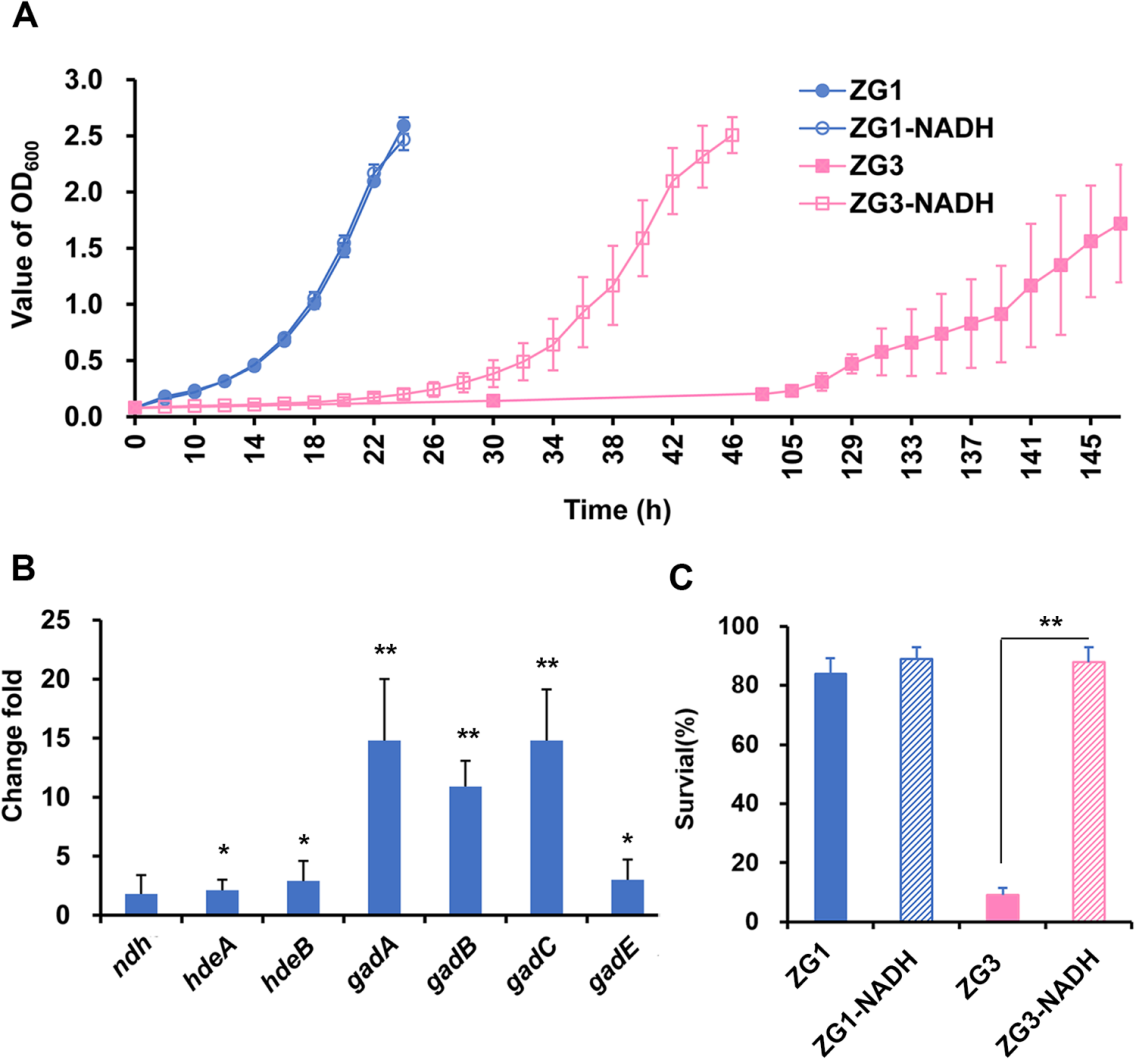
**The effect of NADH on the WT and ZG3 strains.** (A) Growth curves for ZG1 (*icdA*^NADP^, WT) and ZG3 (*icdA*^NADP^Δ*udhA*) with or without NADH supplementation. ZG1-NADH and ZG3-NADH indicate that NADH was added to the culture medium at the beginning. The dashed line represents the long lag phase of ZG3. The growth rate of ZG3-NADH was significantly faster than that of ZG3 (*P*<0.05). (B) Q-PCR results regarding gene expression levels in ZG3-NADH compared with ZG1-NADH. (C) Acid resistance assay for ZG1 and ZG3 cells with or without NADH supplementation. Data are presented as the mean±s.d. of three independent experiments. *P<0.05. **P<0.01.

In order to verify the effect of UdhA on the production of NADH, the concentrations of NADH in ZG1, ZG2, ZG3, ZG4, ZG7, ZG10 and ZG13 were determined by HPLC. The NADH concentrations in all strains were not significantly different when glucose was used the carbon source (Table S1). However, when acetate was used as the carbon source, the NADH concentrations were approximately 8.26, 7.51, 2.63, 6.88, 3.88, 6.82 and 5.82 μmol per gram of cells, respectively (Table 4). The NADH concentration in ZG7 was significantly lower than that of ZG1, which was in accord with the low growth rate of ZG7. In ZG3, the concentration of NADH was the lowest among these strains, about 2.63 μmol per gram of cells (Table 4). It was proposed that NADH may play a key role in ZG3. After the addition of 5 mM NADH to the culture medium at the beginning of culture, the growth rate of ZG1 was nearly the same with or without NADH supplementation (Fig. 4A), whereas the growth rate of ZG3 was significantly increased (*P*<0.01). Furthermore, NADH significantly increased the expression of genes involved in acid resistance, especially the genes (*gadA*, *gadB* and *gadC*) in AR2 (Fig. 4B). The acid resistance assay confirmed that NADH significantly increased the survival rate of ZG3 from 10% to the same level as ZG1 (*P*<0.001), whose survival rate was not affected by NADH supplementation (Fig. 4C). These results demonstrated that UdhA could affect growth rate and survival of ZG3 in a NADH-dependent manner.

**Table 4.**
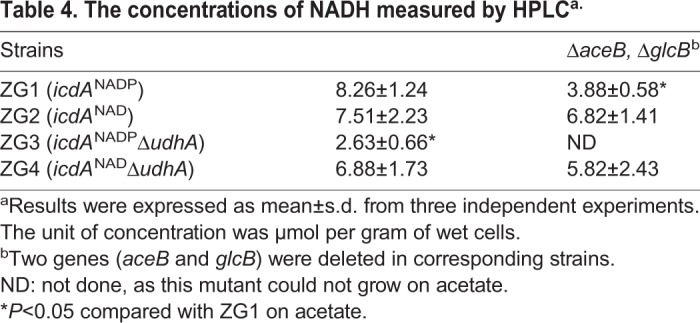
**The concentrations of NADH measured by HPLC^a.^**

## DISCUSSION

Gram-negative bacteria exhibit several adaptations for survival and growth at low pH. The most effective acid-resistance system in *E. coli* is AR2 (Ma et al., 2003; Tucker et al., 2002). Intracellular glutamate is decarboxylated to 4-aminobutyrate and CO_2_, with stoichiometric consumption of protons, by either of two isozymes, GadA or GadB. Intracellular 4-aminobutyrate is then exchanged for external glutamate by GadC. The combined activity of these three enzymes efficiently consumes protons, increasing the intracellular pH to levels compatible with survival (Foster, 2004; Richard and Foster, 2004, 2003). The DNA array data showed that the expression of *gadA* and *gadBC* was lower in ZG3 (*icdA*^NADP^Δ*udhA*) than in wild-type ZG1 (*icdA*^NADP^) during growth on acetate. These results are consistent with the acid resistance experiments. More than 80% of ZG1 and ZG4 cells survived for 1 h at pH 4.9, whereas less than 10% of ZG3 cells survived (Fig. 2). Transforming ZG3 with expression vector, pGadA or pGadBC, improved survival to 35% or 50%, respectively (Fig. 2), whereas transforming ZG1 or ZG4 with the same vectors had little effect (Fig. 2).

In addition to the decarboxylase, other acid resistance genes expressing the periplasmic acid chaperones *hdeA* and *hedB* were downregulated in ZG3 (Table 3). Chaperones provide another adaptation to low pH (Bearson et al., 1997). In *E. coli*, chaperones HdeA and HdeB suppress the aggregation of periplasmic proteins at low pH (Kern et al., 2007). The expression of *hdeAB*, expressed under *gad* operon, is HN-S repressed, RpoS dependent and acid induced (Richard and Foster, 2004). As in an earlier study (Kern et al., 2007), survival improved when *hdeAB* is overexpressed in ZG3 (Fig. 2). Lower expression of *hdeAB* may be caused by lower expression of GadE, which is believed to bind directly to *hdeAB* operon promoter and induce expression. Survival of ZG3 was found to improve dramatically, from about 12% to 66%, when ZG3 cells harbored pUdhA (Fig. 2). These results indicate that UdhA is important to acid resistance through affecting gene expression of the Gad acid resistance system in *E. coli*.

Furthermore, our developing model indicates that UdhA is an important source of NADH during bacterial growth on acetate. The concentration of NADH in ZG3 is quite low. The lower expression of acid resistance genes in ZG3 is attributed directly to a shortage of NADH. Replacing the NADP-specific *icdA*^NADP^ with the NAD-specific *icdA*^NAD^ in ZG4 and the direct supplementary of NADH in ZG3 replenish the NADH pool, increase the expression of acid resistance genes (such as *gadA* and *gadBC*) and improve growth to almost wild-type levels. NADH could increase *gadABC* expression, which may affect *gadE* expression and subsequently regulate GAD operon (Ma et al., 2003). Another possibility of UdhA deletion affecting acid resistance of *E. coli* in acetate is that the low NADH pool could not provide enough production of ATP, which affects the acid resistance system (Sauer et al., 2004; Sun et al., 2011). More experiments are needed to verify these possibilities.

NDH-2, the primary dehydrogenase during aerobic respiration, catalyzes the re-oxidation of NADH, transferring the electrons to ubiquinone-1 (Calhoun and Gennis, 1993; Gyan et al., 2006). NDH-2 is tightly regulated by global transcription factors and links the major catabolic and energy-producing pathways (Jaworowski et al., 1981). The down-regulation of *ndh* in ZG3 might be a mechanism for bacterial adaption to a poor NADH status. The effect of NADH on *ndh* expression was further confirmed by the NADH supplementation experiment. The concentration of NADH in ZG3 cells measured via HPLC was the lowest among all of the tested strains, whereas the supplemental NADH restored the expression of *ndh* in ZG3 to a level similar to that in the wild-type strain ZG1 cells (Fig. 4B). Thus, UdhA is an important source of NADH. The depletion of the NADH pool, as observed in ZG3, reduces the expression of respiratory chain genes and, hence, growth.

Even though *udhA* was deleted in ZG4 and ZG13, the growth rates of these two strains were similar to the corresponding strains (ZG4 to ZG2; ZG13 to ZG10). The concentration of NADH did not differ significantly among these four strains. These results illustrate that in the *icd^NAD^* background, UdhA does not play an important role in supplying the NADH pool. By contrast, *udhA* was very important in the wild-type (ZG1-*icd^NADP^*) background, as the growth rate of ZG3 and NADH concentration in ZG3 were significantly lower compared with ZG1, and it was important for bacterial adaption to acid stress. Taken together, the soluble transhydrogenase, UdhA, is an important source of NADH of *E. coli* growth on acetate, and plays roles in affecting the expression of genes involved in the Gad acid resistance system under acetate stress.

## MATERIALS AND METHODS

### Materials

Restriction enzymes were purchased from New England Biolabs (Beverly, USA). Plasmids and genomic DNA were extracted using Wizard^®^ purification kits from Promega (Madison, USA). Herculase™ enhanced DNA polymerase (Stratagene, California, USA) and PrimeStar™ HS DNA polymerase from TaKaRa (Dalian, China) were used for PCR. The GeneChip^®^ *E. coli* genome 2.0 array was employed for genome-wide expression profiling (Affymetrix, California, USA). The SV Total RNA Isolation System for total RNA extraction was purchased from Promega, and Superscript II reverse transcriptase was used for reverse transcription (Invitrogen). The LightCycler-FastStart DNA Master SYBR Green I Kit for RT-PCR was obtained from Roche Applied Science (Indiana, USA). Oligonucleotides were synthesized by Sangon (Shanghai, China).

### Strains and plasmids

All experiments were conducted with strains derived from *E. coli* MG1655 (ATCC. 700926) (Table 1). Two strains, ZG1 (*icdA*^NADP^) and ZG2 (*icdA*^NAD^), containing NADP^+^-dependent isocitrate dehydrogenase (NADP-IDH) and an engineered NAD^+^-dependent IDH (NAD-IDH), and two strains in which *udhA* was deleted, ZG3 (*icdA*^NADP^Δ*udhA*) and ZG4 (*icdA*^NAD^Δ*udhA*), which also contained NADP-IDH and an engineered NAD-IDH, were constructed previously (Zhu et al., 2005). All other strains were constructed specifically for this study. The plasmids used in this study are listed in Table S2.

### Culture conditions

Lysogeny broth (LB), Super Optimal broth (SOB), Super Optimal broth with catabolite repression (SOC) and MOPS-based minimal medium were prepared as previously described (Zhu et al., 2005). MOPS-based minimal medium (250 ml, pH 7.4) included 20.925 g MOPS, 1.800 g Tricine, 0.007 g FeSO_4_·7H_2_O, 1.275 g NH_4_Cl, 0.120 g K_2_SO_4_, 7.300 g NaCl, 0.137 g CaCl_2_, 0.124 g MgCl_2_, 2.5 mg Vitamin B1. LB and SOB media were supplemented with 100 μg ml^−1^ ampicillin, 10-15 μg ml^−1^ tetracycline, 30 μg ml^−1^ kanamycin, or 20 μg ml^−1^ chloramphenicol, as required.

### Growth rates

Strains were grown in 25 ml of MOPS-based minimal medium containing 2% glucose or 2% acetate as a carbon source in a 250 ml flask in an orbital shaker (200 rpm) at 37°C. Samples were taken every 45 min (glucose) or 2 h (acetate), and culture densities were determined spectrophotometrically at 600 nm (UV-2102 spectrophotometer, UNICO Co. Ltd). Growth rates were calculated using linear regression on semi-logarithmic plots of OD_600_ against time and processed by JMPIN software. The growth rates were from at least three independent experiments.

### RNA extraction

RNA was isolated and purified by using the SV Total RNA Isolation System according to the manufacturer's instructions. After purification, genomic DNA was removed by treatment with RNase-free DNase I (Promega), followed by RNA repurification with an RNeasy column. The quantity and purity of RNA were determined by measuring *A*_260_ and the *A*_260/280_ ratio, respectively. Purified total RNA was precipitated with ethanol and stored at −80°C until needed.

### Microarray analysis

All protocols were conducted as described in the Affymetrix GeneChip Expression Analysis Technical Manual and produced by CapitalBio Corporation (Beijing, China). The GeneChip^®^ *E. coli* genome 2.0 array, containing 20,366 genes, was used to study the differential expression profiles of strains ZG2 (*icdA*^NAD^), ZG3 (*icdA*^NADP^Δ*udhA*) and ZG4 (*icdA*^NAD^Δ*udhA*), compared with ZG1 (*icdA*^NADP^, wild-type MG1655). Total RNA (10 µg) was reverse transcribed with random primers using SuperScript II reverse transcriptase. The resulting cDNA was fragmented into sequences ranging from 50–200 bp and labeled at the 3′ termini using the GeneChip^®^ DNA Labeling Reagent (Affymetrix, P/N 900542). Biotin-labeled cDNAs were hybridized to an Affymetrix GeneChip^®^ *E. coli* genome 2.0 array at 45°C for 16 h at 60 rpm using a Hybridization Oven 640 (Affymetrix). The array was washed and stained on an Affymetrix Fluidics Station 400. The microarray was scanned using the default settings on a GeneChip Scanner 3000. The scanned images were analyzed with GeneChip Operating Software (GCOS 1.4). Intensities were scaled using dChip software to allow direct comparisons between strains. All experiments were performed in two biological replicates, and transcripts showing a ≥twofold change in expression and *P*<0.05 were considered significant. The full microarray data are accessed by NCBI BioProject (PRJNA382597).

### Real-time PCR

Real-time PCR was performed to validate the microarray data. The cDNAs used for microarray analysis were used as the templates in Real-time PCR. The LightCycler-FastStart DNA Master SYBR Green I Kit was employed for real-time RT-PCR. The RT-PCR assays were performed using an initiation step of 10 min at 95°C, followed by 40 cycles of 15 s at 95°C, 5 s at 58°C and 15 s at 72°C, with fluorescence data collection at 76°C using LightCycler Software Version 3.5. Controls were used to ensure that there was no contaminating DNA in the RNA sample. Quantification of the *ndh* and *icdA* genes relative to a housekeeping gene (16S rRNA) was performed using a mathematical model by Paffl (Pfaffl, 2001), which accounts for real-time RT-PCR efficiencies and the crossing points for the transcripts of each sample.

### Plasmid constructions

The downregulated genes (*hdeAB* operon, *gadA*, *gadBC* operon, *gadE*) identified with the microarrays and the *udhA* gene were amplified via PCR using *E. coli* MG1655 genomic DNA as a template. The forward primers contained a *Hind*III or *Xba*I site, and the reverse primers contained an *Xho*I or *Hind*III site (Table S3). The PCR products were digested and ligated into digested pBluescript SK(+) II (pBlue) and then transformed into ZG1, ZG3 and ZG4 cells. The plasmid constructs (Table S2) were confirmed through DNA sequencing.

### Acid resistance assay

The ability of the strains to survive acid stress was evaluated as previously described (Abdul-Raouf et al., 1993; Castanie-Cornet et al., 1999; Sainz et al., 2005). Cells were grown in MOPS-acetate medium with antibiotics, 1 mM IPTG or 1.5 mM glutamate, as required. After the bacteria were inoculated into acetate medium, the cells were grown exponentially to an *A*_600_ of 0.6 and then diluted 1:100 into fresh acetate medium, pH 4.9, followed by incubation for 1 h at 37°C. The acid challenge was stopped through dilution. The samples were serially diluted and plated onto LB plates. The percentage of surviving cells was estimated from the number of colonies at 1 h relative to the number at time zero. Each experiment was repeated at least three times.

### Construction of *allB*, *gcl*, *aceB* and *glcB* deletions

The genes were disrupted via the method of Datsenko and Wanner (Datsenko and Wanner, 2000). Briefly, primers for the deletions (Table S4) were used to amplify the kanamycin resistance gene from pKD13 (Datsenko and Wanner, 2000). Strains harboring pKD46 were grown in SOB medium containing 200 μg ml^−1^ ampicillin and 10 mM L-arabinose at 30°C to mid-log phase. Linearized PCR products containing *kan* and flanking sequences on either side of *allB*, *gcl, aceB* or *glcB* were introduced into competent cells via CaCl_2_ transformation. Cells, shocked at 42°C and recovered at 37°C in SOC, were spread on LB plates containing kanamycin. The desired disruption was confirmed via sequencing PCR-amplified regions spanning 0.5∼1 kb of the flanking DNA on either side of the inserted *kan* gene. Next, the *kan* genes were eliminated from the strains, and ampicillin-resistant transformants carrying pCP20 were isolated and grown at 42°C on LB plates (Datsenko and Wanner, 2000). Colonies were screened for sensitivity to ampicillin and loss of pCP20. Multiple deletions were constructed sequentially. Finally, *icd* alleles (*icdA*^NADP^, which encodes NADP-IDH, or *icdA*^NAD^, which encodes NAD-IDH) were introduced via P1 cotransduction with adjacent *tet^r^* cassettes. All together twelve strains were constructed (Table 1), four with single deletions (ZG5, ZG6, ZG8, ZG9), five with double deletions (ZG7, ZG10, ZG11, ZG12) and two with a triple deletion (ZG13). Chromosome-integrated regions were sequenced to ensure that no other mutations had been inadvertently introduced during construction.

### NADH extraction and quantification through HPLC

To extract NADH, 250 ml cell cultures were centrifuged at 4600 rcf for 5 min and resuspended in extraction buffer [1 ml of 0.5 M Tris-HCl, pH 7.0 containing 1 mM EDTA and 1 ml of methanol (Heuser et al., 2009)]. After the addition of 2 ml of chloroform, NADH extraction was performed by shaking the tube for 1.5 h at 25°C. Prior to extraction, 1 mM and 100 mM NADH was added to a parallel sample as an extraction control. The separation of NADH in the aqueous phase was carried out via centrifugation for 5 min at 3200 rcf to separate the aqueous phase from the chloroform phase. The aqueous phase was filtered through a 0.22 µm filter to remove denatured protein. HPLC quantification of NADH was performed with a Prominence LC20 (SHIMADZU) on a column (VP-ODS C18 15X0.2). Buffer A (pH 6.0) contained 0.1 M potassium phosphate buffer and 4 mM tetrabutylammonium hydrogen sulfate, and buffer B (pH 7.5) contained 70% buffer A and 30% methanol. The two buffers were applied by mixing 100% buffer A and 0% buffer B at the beginning, followed by 70% buffer A and 30% buffer B after 12.5 min, 40% buffer A and 60% buffer B after 25 min and 0% buffer A and 100% buffer B after 32.5 min. The total separation time was 45 min. The flow rate was 0.2 ml min^−1^.

### Statistical analysis

Statistical analyses were performed with the two-tailed Student’s *t*-test using Stata statistical software. The *P* value <0.05 was considered as statistically significant.

## Supplementary Material

Supplementary information
