# Supplementary material for: The soluble transhydrogenase UdhA affecting the glutamate-dependent acid resistance system of Escherichia coli under acetate stress
Source: Biol Open. 2018 Sep 15;7(9):bio031856. doi: 10.1242/bio.031856 (PMC6176936; doi:10.1242/bio.031856)
Supplement: Supplementary information [file biolopen-7-031856-s1.pdf]

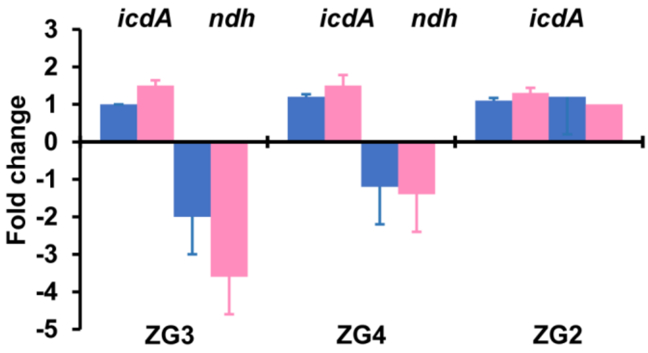

Fig. S1. Quantitative RT-PCR verification of microarray data. The expression levels of the *icd* and *ndh* genes in ZG1 (*icdA*^{NADP}, WT), ZG2 (*icdA*^{NAD}), ZG3 (*icdA*^{NADP}/Δ*udhA*) and ZG4 (*icdA*^{NAD}/Δ*udhA*) were estimated through quantitative RT-PCR analysis. The blue bars (■) represent the relative change estimated based on microarray data. The pink bars (■) represent the relative change estimated using RT-PCR. Data are presented as the average of two independent experiments.

Table S1. The concentrations of NADH measured by HPLC

Strains	Glucose [a]	
		Δ*aceB*, Δ*glcB* [b]
ZG1 (*icdA*^{NADP})	9.58±6.67	7.15±2.96
ZG2 (*icdA*^{NAD})	6.04±2.27	5.06±0.85
ZG3 (*icdA*^{NADP}Δ*udhA*)	4.76±5.89	ND
ZG4 (*icdA*^{NAD}Δ*udhA*)	5.65±2.21	6.31±0.75

[a] Results were the mean ± S.D. of three independent experiments. The unit of concentration was μmol per gram of wet cells.

[b] Two genes (*aceB* and *glcB*) were deleted in corresponding strains.

ND: not done

Biology Open • Supplementary information

Table S2. List of plasmids

Plasmids	Genotype	reference
pHdeAB	*hdeAB* operon (acid resistance proteins) in pBluescript SK(+) II	This study
pGadA	*gadA* (glutamate decarboxylase isoenzyme) in pBluescript SK(+) II	This study
pGadBC	*gadBC* operon (glutamate decarboxylase isoenzyme and glutamate/γ - aminobutyrate transporter) in pBluescript SK(+) II	This study
pGadE	*gadE* (acid-induced regulator) in pBluescript SK(+) II	This study
pUdhA	*udhA* (soluble transhydrogenase) in pBluescript SK(+) II	This study
pGcl	*gcl* (glyoxylate carboligase) in pBluescript SK(+) II	This study
pGlxR	*glxR* (tartronate semialdehyde reductase) in pBluescript SK(+) II	This study
pHyi	*hyi* (hydroxypyruvate isomerase) in pBluescript SK(+) II	This study
pAllB	*allB* (allantoinase) in pBluescript SK(+) II	This study

Biology Open • Supplementary information

Table S3. Oligonucleotides used for plasmid constructions

Primer	Oligonucleotide sequence (5' to3') [a]	Restriction enzyme
pHdeAB-N	CGCGATCGAAGCTTTTCACTTTATAGTTGAG	*Hind*III
pHdeAB-C	GTACTAGTCTCGAGTTTTTACTTGCACCTCATTAA	*Xho*I
pGadA-N	CACGAGAAGCTTTGCCTTCAAATAAATTTAAGGAG	*Hind*III
pGadA-C	GTCAGCACTCGAGCAACACGATGAATAGACAGC	*Xho*I
pGadBC-N	GACGTGACAAGCTTCTTTGCACTTGCTTACTTT	*Hind*III
pGadBC-C	GTGATGACTCGAGCAATACGACAGCAAGCA	*Xho*I
pGadE-N	CAGCACAAGCTTGAAACGATAACGGCTAAG	*Hind*III
pGadE-C	GTCAGCACTCGAGAATGAAATCAGCATCTG	*Xho*I
pUdhA-N	CGCAGCACTCTAGACATGCCACATTCCTACGATT	*Xba*I
pUdhA-C	CAAACACTCGAGTTAAAACAGGCGGTTTAAACCGTTTA	*Xho*I
pGcl-N	GATCCTGCAGATGGCAAAAATGAGAGCCGTTGACG	*Pst*I
pGcl-C	TACGAAGCTTTTATTTATCTCCCTTATTCATAGTG	*Hind*III
pGlxR-N	GTCGACTGCAGATGAAACTGGGATTTATTGG	*Pst*I
pGlxR-C	GCATGAAGCTTTTTATTGCGGGTATCAGG	*Hind*III
pHyi-N	GTCATCTGCAGTAAGGGAGATAAATAATGTTAC	*Pst*I
pHyi-C	GATCGAAGCTTAACTTAGCGGCCTAAAAAAGCATTG	*Hind*III
pAllB-N	CTAGCAAGCTTACAAGGAGTTTGTTATGTCTTTTG	*Hind*III
pAllB-C	CTGCACTCGAGCCAGATTACTGCTGATGTTTAAG	*Xho*I

[a]Cutting sites for restriction enzyme are underlined.

Table S4. Oligonucleotides used for constructing recombinant strains

Primer	Oligonucleotide sequence (5' to3')
Δ*allB*-Fa-N	CGGTAATTGGTGTCATGATGGCGCATTAT
Δ*allB*-Fa-C	CCGGAATTAATTCTCATGTTTGACAGCTTATCACTATAAAACTCCTTGTAAAATA
Δ*allB*-Fb-N	AGCAGCTCCAGCCTACACAATCGCTCAACTAAGAAATCCTTAAACATCAGCAGTAATCT
Δ*allB*-Fb-C	TGTCCCGCGCGATGCTTCACCAAGGGT
Δ*gcl*-Fa-N	AACTACGCTTGTGGATATGCCCACCTTG
Δ*gcl*-Fa-C	CCGGAATTAATTCTCATGTTTGACAGCTTATCACTATAACATTTTATTCCTACCTCTATTT
Δ*gcl*-Fb-N	GAAGCAGCTCCAGCCTACACAATCGCTCAACTAAGAATGCTGGTCAACAACGCTTATC
Δ*gcl*-Fb-C	AGGTTTCAGTCGGTGCGTCCGCTGCGTTATCG
Δ*aceB*-Fa-N	TCAGCACCTTACCTCAGGCACCTTCGGG
Δ*aceB*-Fa-C	CCGGAATTAATTCTCATGTTTGACAGCTTATCACTATAATCACCAGCTCAGTCAGAAATTCTA
Δ*aceB*-Fb-N	GAAGCAGCTCCAGCCTACACAATCGCTCAACTAAGAAGTATTCAACGACATTCTCGGCTC
Δ*aceB*-Fb-C	GCGGCATCGTCAAAACGCCCCTGGGA
Δ*glcB*-Fa-N	GCTGTTGCCGATGACGGCGGTCATCTGCTGG
Δ*glcB*-Fa-C	CCGGAATTAATTCTCATGTTTGACAGCTTATCACTATAAGGCTCTGGGTTATGGTTTGACT
Δ*glcB*-Fb-N	CGAAGCAGCTCCAGCCTACACAATCGCTCAACTAAGAAGACGCCTTGGATCAAAGCCTACG
Δ*glcB*-Fb-C	CGCACCACGTACCCCAGAATCCCCTGCACGTTGT
kan-N	TTATAGTGATAAGCTGTCAAACA
kan-C	TTCTTAGTTGAGCGATTGTGTAG

Fa means that the homologous sequence used for homologous recombination was located at the upstream of the target gene; Fb means that the homologous sequence used for homologous recombination was located at the downstream of the target gene.

Biology Open • Supplementary information
